# Chronic Illness and Income Diversification in Rural China

**DOI:** 10.3390/ijerph18073350

**Published:** 2021-03-24

**Authors:** Wenmei Liao, Jiawei Wang, Ying Lin, Yao Wang

**Affiliations:** 1 School of Economics and Management, Jiangxi Rural Revitalization Strategy Research Institute, Jiangxi Agricultural University, Nanchang 330045, China; liaowenmei@126.com (W.L.); wang13319407091@126.com (J.W.); 2 School of Economics and Finance, Xi’an Jiaotong University, Xi’an 710061, China; 3 Department of Human Resources and Social Security of Shaanxi Province, Xi’an 710000, China; 11320020@zju.edu.cn

**Keywords:** health, off-farm, livelihood strategies, diversification

## Abstract

Off-farm diversification offers an important pathway out of poverty while health-impaired rural farmers can hardly seize the opportunity in developing countries. This paper investigates how chronic illness shapes livelihood structure and income generation in rural China. Our sample consists of 3850 rural households in Southern China and we rely on instrumental variable regressions to identify causal effects. We find that farmers with chronic illness tend to diversify towards local off-farm employments, rather than migrants, since local off-farm employments are more likely to act in a strategically complementary way to farming. Further analysis shows that income returns of diversification tend to be substantially higher for the health-impaired. While the relationship between diversification and income presents a conventional inverted U shape for the healthy, it is best categorized as upward sloping with diminishing marginal effects for farmers with chronic illness.

## 1. Introduction

Diversification is a dominant livelihood strategy for rural households in developing countries, of which many derive a large part of their income diversifying towards off-farm activities and migration [1]. Off-farm employments may already account for as much as over 30% of the rural working-age individual population in Sub-Saharan Africa, and 75% in China, and it seems to be growing in importance [2,3]. While a steady trend towards off-farm employments is expected to alleviate and reduce poverty in rural populations during the process of economic and structural transformation [4], the health dimension is often neglected in analyses of off-farm diversification and poverty reduction. An influential study pointed out that people in good health are more likely to be economically productive [5]. Another more recent study found that better health increases the probability of labor force participation in all age groups [6]. Moreover, health problems contribute towards an individual not attaining his/her potential income frontier [7]. It still remains unclear how off-farm employments of the rural population are constrained by chronic illness. Understanding their health status and livelihood responses can guide effective policy interventions targeted at alleviating rural poverty and vulnerability.

This paper presents a systematic analysis on income returns to diversification in rural China with a particular focus on its relationship with chronic illness. The motivations for the paper are as follows. First, the paper adds to the literature by examining the livelihood responses of the rural farmers to ill health from a portfolio perspective. A wide range of literature focuses on the effect of health on wage employment and related labor supply, e.g., [6,7,8]. Rather less attention has been paid to the fact that whereas farming remains the occupation of choice for most rural households [9], the proportion of self-employment has increased in the developing world over recent decades [10]. This paper accounts for different strategies of livelihood arguing that portfolio adjustments will help develop further insights into the interaction between ill health, livelihood choice and income in rural China. Specifically, we focus on chronic illness-induced heterogeneity in livelihood choices and the possibility of strategy complementarity between farming, local off-farm employments and/or migrants.

Second, this paper also adds to the literature by examining income returns to diversification and whether the relationship varies by health status. Studies present evidence on the degree of livelihood diversification with respect to labor asset while the results are quite mixed and even opposite across research areas, e.g., [11,12,13]. We further extend the scope of labor asset to its quality level. A quadratic specification is adopted to investigate whether a U-shaped, an inverse U-shaped or another pattern for diversification and income exists and whether the pattern differs with respect to health status of the household breadwinner in rural China. The scarce literature on this issue in the health dimension leaves the nature of this relationship as an open empirical question to be tested. 

Third, previous studies have analyzed either the determinants of household income diversification or the relationship between diversification and poverty reduction in rural China, e.g., [14,15,16]. These studies use discrete indicator variables for different types of portfolios and/or overlook the potential endogeneity of diversification. A brief review of our data revealed that diversification to off-farm employments is a norm in rural China, which is also typical in the rural areas of most developing countries such as Indonesia and Nigeria [12,13]. Following recent studies on livelihood diversification, e.g., [17,18], a continuous index is adopted that encompasses both the magnitude and the number of income sources. Furthermore, one of the advantages of our field survey is that non-agricultural wealth information is available the year before, which allows us to address the potential endogeneity of diversification using predetermined wealth variables. 

The reminder of the paper is organized as follows. Section 2 presents stylized facts regarding health status and rural livelihood and introduces the background of China’s rural public health system. Section 3 builds generic frameworks for the econometric analyses and summarizes the study area and data. Section 4 serves to present regression results for the effect of chronic illness on livelihood choices and income returns to diversification. We interpret the relationship between diversification and income and discuss how chronic illness contributes to the formation of the relationship. Section 4 concludes with implications for policy and further research. 

## 2. Literature Review

### 2.1. Existing Evidence at the Micro and Macro Levels

In the rural development context, income diversification mostly refers to the desirability of expanding outside agricultural activities to off-farm industries as a dynamic adaptation strategy which either enhancing existing security and wealth or reducing vulnerability and poverty [4,13,19]. Most rural households have multiple off-farm income sources including off-farm wage work in agriculture, wage work in non-farm activities, rural non-farm self-employment, and remittances from urban areas and from abroad [4]. While traditional wisdom depicts diversification as a risk coping and management strategy, a recent study points out that, if off-farm income opportunities are available and functioning financial markets exist, livelihood or production portfolio (i.e., a collection of agricultural and off-farm activities) is less likely to depend on risk preferences as activities realize strategic complementarities [20].

Ill health, a condition in which some disease or impairment of function is present but is usually not as serious as curtailing activity, provides both push and pull reasons for diversifying. The former refers to involuntary and distressful diversification as a rural family loses the ability to continue to undertake strenuous agricultural activities [4]. Empirically, 67% of the farms have ceased agricultural production five years after an accident occurred, and 44% of them were no longer live on the farms in the United States [21]. In Norway, farmers’ health status plays a statistically significant role in explaining the variance of agricultural inefficiency [22]. Employing stochastic frontier regression techniques, similar results were found in Ethiopia [23]. Some researchers evaluate the agricultural productivity effects of health focusing on specific health problems such as schistosomiasis and malaria [24,25]. Conceptually, empirically literature verifies lower labor supply and productivity in farming with ill health in both low-income and developed settings. 

By contrast, ill health is a pull factor to off-farm diversification as a voluntary and proactive choice for healthcare. Farmers are consistently identified as being at high risk for injury and fatality [26]. However, the healthcare systems in less developed countries, like Chile, India and China, are generally government-controlled that tend to be poorly funded and held to lower standards than privatized healthcare [27]. Since private health insurance plans offer a potential alternative to insure against the cost of illness [28,29], many farm households in the United States have family members working off the farm for fringe benefits such as employer-sponsored healthcare [30]. Mishra and Chang suggest that there is a significant and negative effect of off-farm work decisions of the U.S. farm operators and spouses on healthcare expenditures [31]. 

### 2.2. Public Health System in Rural China

In addition to the push and pull reasons of ill health for diversifying, a more subtle third channel in China relates to the migration barrier intensified by its public healthcare system. In rural China, an estimated 99% rural population are covered under government-sponsored public health insurance, namely the New Rural Cooperative Medical Scheme (NRCMS) [32]. This employment-delinked health insurance helps stabilizing protection against unexpected losses of health problems, which would depress off-farm labor force participation especially that of the health-impaired population [33]. Moreover, reimbursement of the NRCMS adopts a grading medical system. That is, the reimbursement rates of inpatient treatment decrease literally with distance to the rural residence. There is a 10 to 20 percent decrease of the reimbursement rates if a rural patient chose a county-level hospital instead of the community (town-level) health service institutions and a further 0 to 15 percent decrease for a superior or out-of-province hospital. Anticipating increasing medical costs with distance to the place of residence, health-impaired rural population may prefer to choose farming and local employments instead of migrants ex ante as a coping response to health shocks.

## 3. Econometric Analysis

### 3.1. Livelihood Choice Model

According to people’s main occupations and profession in the economy [34], we propose the following joint choice model to study the effect of ill health on rural livelihood strategies.

{(1a)Pr(farmit=1|Z)=Φ(ρIjt+αilli+τXit)(1b)Pr(localit=1|farmit,Z)=Φ(λfarmit+βilli+φXit)(1c)Pr(outit=1|farmit,Z)=Φ(νfarmit+γilli+ϕXit)

where *farm_it_* is a dummy variable denoting whether farming is an income source for household *i* in year *t*. Farming is defined as the production or gathering of unprocessed crops or livestock or forest products from natural resources. *local_it_* and *out_it_* are dummy variables denoting whether off-farm employments are sources of income for household *i* in year *t.* Off-farming employments include all activities away from one’s own property regardless of sectoral or functional classification and can be wage or self-employment. We especially distinguish participation in local off-farm employments (*local_it_*, i.e., within county employments) from migrants (*out_it_*, i.e., out-of-county jobs). The relationships between farming probability and off-farming participations are assessed in Equation (1b,c). Our promise is that if the health-impaired farmers are constrained to the resident (as by the reimbursement system of the NRCMS), local off-farm employments will be a strategic choice complementary to farming geographically and economically. Thus, a significant and positive λ is anticipated in Equation (1b) whereas a significant but negative ν is in Equation (1c).

The endogeneity problem may rise when an unobserved or omitted variable, such as ability, is confounding both farming and non-farming participations in Equation (1b,c). We therefore include an instrumental variable for *farm_it_* in Equation (1a) to achieve consistent estimation of λ and ν. As always, the instrument needs to be an extra covariate that is associated with farming participation while not being directly related to local off-farm and migrant decisions [35,36]. The instrument, poverty at the village level (*I_jt_*), is a dummy variable denoting whether village *j* is designated as a poor village by the local government, which we argue fulfil the required criteria. A government-designated poor village in China has an average disposable income of all rural populations as well as that of the poorest fifth under a proactive regional poverty standard. The instrument indicates regional differences in basic living standards, so all else is equal, and we expect a household resident in a poor village to have a higher probability of working on farming for subsistence needs. Besides, regional poverty may affect off-farm labor participation indirectly through intra-community social and economic resources and networks [37].

A predetermined dummy variable is adopted to denote the health status of household *i*. *ill_i_* equals 1 if the breadwinner of the household had been diagnosed with at least one chronic disease before the year of interview, and 0 otherwise. Control measures in vector *X_it_* are a set of policy-specific indicators, household demographic characteristics and geographical dummies. For demographic-specific factors, we include the age of the breadwinner, household size, number of youths (age under 16) and area of cultivated land. For policy-specific factors, we include subsistence allowance and agricultural subsidy received by the household. 

### 3.2. Diversification Returns Model

Given that the level of livelihood diversification of health-impaired farmers may differ with their counterparts, we seek to further identify different income returns to diversification with respect to health status. The effects of diversification on income by health status is estimated through the following model:

(2) $$income_{it}=\delta_{1}di_{it}+\delta_{2}di_{it}^{2}+\delta_{3}di_{it}*ill_{i}+\delta_{4}di_{it}^{2}*ill_{i}+\zeta ill_{i}+\xi X_{it}+\omega_{it}$$

where *di* is a normalized Herfindahl–Simpson income diversification index that equals one minus the normalized Herfindahl–Simpson concentration index. The index compensates for the effect of evenness and dominance by capturing dimensions of both the distribution of income earning from different sources and the number of income sources:

(3) $$di_{it}=1-\frac{\sum_{1}^{N}s_{ikt}^{2}-\frac{1}{N}}{1-\frac{1}{N}},\;0\leq di_{it}\leq 1$$

where *s_ikt_* represents income share of the *k-th* livelihood source for household *i*. The index falls to the minimal value of zero with dependence on a single livelihood source and approaches the maximal value of one with full diversification of income.

A squared term of *di* is included to identify whether there is a linear, a U-shaped or an inverse U-shaped response of income to diversification. Health shocks experienced by households and their interaction with diversification are included to estimate the resilience capacities of rural livelihood diversification strategies. As such, diversification in the model is a capacity indicator that may facilitate recovery following the health shock. δ_1_ and δ_2_ may be interpreted as the effect of household level capacities on welfare, as indicated by income, without health shocks. ζ is interpreted as the effect of chronic illness on income without household level resilience capacities, and δ_3_ and δ_4_ are the combined effects of household level resilience capacities and health shocks in mitigating or aggravating the impact of shocks.

Income offers a measure of direct interest because of its clear interpretation as a welfare outcome. The difficulty is to distinguish the direction of causality between diversification and income. To reduce potential endogeneity of diversification, we use initial household wealth indicators, i.e., non-productive asset and household debt, as instruments within a two stage least square framework. Studies verify that better-off households are more diversified in Nigeria and Ethiopia [38,39]. Non-agricultural asset is measured as the total number of household appliances and electronic equipment irrespective of unit. Household debt is defined as the combined liabilities of all people in a household. Furthermore, values before the year of interview are adopted because of the permanent and ex ante nature of initial wealth as a stock is less subject to endogeneity than diversification as a flow. The set of control variables *X_it_* is defined the same as those in model (1).

### 3.3. Study Area and Data

Our econometric analyses rely on a data set with independent rural households in Jiangxi Province, southeast of China. Although developing quickly, the average disposable income of rural residents of Jiangxi province (RMB 12,137 per capita in 2016; RMB is the currency code of China) is still under the national average (RMB 12,363 per capita). Rice remains the dominant crop in agricultural production. In December 2016, our research team conducted a large-scale field survey on rural transformation and poverty reduction in Jiangxi. A sample of 4000 rural households were randomly selected from a total number of 5.37 million and face-to-face interview is adopted in accordance with the prepared questionnaire. The study purpose was told to participants before the interview and participations could withdraw at any point during the interview. The participations are household breadwinners and none of them had asked permission from other household members before the interview. We caution that our survey may still suffer from some coercion because of poverty and/or low literacy while the variations are controlled in models (1) and (2). After deleting samples with incomplete information or missing value, 3850 complete interviews were received, with a response rate of 96.25%. 

Variables in models (1) and (2) are reported in Table 1. As representative measures of rural income from different sources, we use farm income, local off-farm income, migrating income and other income. It should be noted that agricultural and forest products consumed by the household are included in farm income. Migrating income specifically refers to the net benefits of family labors both working and resident permanently or temporarily at a geographically new location which is typically in urban areas. Other income is the sum of property income from renting out real estate, arable land and forestland and net interests from interest-bearing deposits, loans and securities.

A relatively high percentage of rural households, 79.61%, state that their breadwinners have been detected with at least one kind of chronic diseases before 2014. One of the reasons is that we adopt a rather wide definition of chronic disease and the ones with virtually weak work limitations are also included such as high blood pressure, high cholesterol, arthritis, diabetes and migraine headaches. Table 2 illustrates the variation of demographic characteristics and livelihood strategies across health status of the household breadwinner. It reveals that chronic illness-bothered households also differ in main demographic characteristics such as age of the breadwinner, household size and number of youths. Health-impaired households receive an average higher subsistence allowance from the governments. Tentatively, higher participation in local off-fam employments and lower migration probability go along with health-impaired rural farmers. While there is no difference in the extent of diversification as indicated by the normalized Herfindahl–Simpson index, per-capita income declines considerably for households with a health impaired breadwinner. In what follows, we analyze the variation in more depth to identify the partial links between cross-household livelihood differences in the extent of health status.

## 4. Estimation Results

### 4.1. Instrument Validity

The robust identification of models (1) and (2) hinges on our instruments. This section first explores validity of the instruments before the paper’s main punchline. Column (1)–(6) of Table 3 reports probit and logit estimates for each of the livelihood choices, with coefficients converted to average marginal effects. Both methods report very similar results. Rural household residents in a relatively poor village have a significantly higher probability engaging in farming by 4.5 percentage points. That is, the instrument of model (1) appears to significantly and nontrivially affect the likelihood of farming. However, there is no formal test of instrument excludability for a trivariate recursive model. We therefore test the probit/logit marginal effects of village poverty on local off-farming and migrating probabilities, respectively. Results in columns (2)–(3) and (5)–(6) indicate that village poverty exerts insignificant influences on both likelihoods, suggesting plausible exogeneity with respect to off-farm employments. 

Both Wooldridge’s [40] robust score test and the robust regression-based F test reject the null hypothesis that diversification is exogenous at the 1% significance level (Table 4, column (10)). Therefore, we are inclined to treat diversification as endogenous in the income equation. As expected, rural households with more non-agricultural assets and fewer debts significantly diversify their livelihood strategies. Instrument excludability is tested using the Wooldridge’s [40] robust score test of overidentifying restrictions. The insignificant test statistic (Table 4, column (11)) indicates that the instruments are uncorrelated with the structural error term.

### 4.2. Livelihood Strategies

The main results of estimating the livelihood choice model with and without equation correlation are reported in Table 3. The coefficient estimates for the single equation Probit and Logit models are reported in the left columns, followed by the seeming unrated estimates. A diagonal disturbance covariance matrix is incorporated to the seeming unrated regression to include the correlation between equations of model (1). The Breusch–Pagan test of independence indicates that we can reject the hypothesis that equation correlation is zero. Specifically, there is quite a large correlation (−0.449) of the residuals in local off-farm employments and migration. The residual correlation of farming between local off-farm employments and migration are both weak, i.e., −0.004 and 0.001, respectively. Hence, relatively small differences are obtained between the Probit/Logit estimates and the seeming unrelated regression.

Mostly independent of the specification considered, the results lend strong support to our key hypothesis that cross-household heterogeneity in health status of the breadwinner (and other demographics and subsidies) is able to systematically explain differences in rural livelihood choices. The effects of chronic illness are heterogeneous across strategies: for farming and off-farm employments, we find the expected positive relationships in all models, with similar marginal effects of chronic illness on farming and non-farming probabilities. While the Probit and Logit models point towards a negative and significant link between chronic illness and the probability of migration, the seeming unrelated trivariate model finds a negative but insignificant relationship.

The conjecture that a rural household’s off-farming diversification strategies vary with farming decisions is strongly supported by the trivariate regression, where we obtain highly significant coefficients for the farming parameter in the local off-farming and migrating equations. In substantive terms, this implies that a rural household would have around a 0.08% higher probability working off-farming locally if it had an average 1% higher probability working on-farm. In the case of migrating, we obtain a significantly negative parameter, indicating about a 0.15% lower migrating probability with an average 1% higher farming probability. Along with statistical significance, the estimates for the average marginal effects of farming participation are economically meaningful: local off-farm jobs are more competitive compared to urban migration for smallholders who had chosen farming as one of their main livelihood strategies. 

Since they account for correlations between livelihood strategies, the seemingly unrelated regression results provide richer information on livelihood responses to illness compared to the Probit and Logit results. In particular, the expanded set of estimators can be used to discriminate between the direct, indirect and total effects of a change in explanatory variables on each of the livelihood choices. The total effect revealed by the Probit and Logit estimates in columns (2)–(3) and (5)–(6) represents the overall impact of an explanatory variable on off-farm employments. The size of this total effect is virtually the sum of a direct effect calculated in columns (8) and (9) and an indirect effect, which can be derived multiplying its effect on farming probability (in column (7)), by the average marginal effect of farming participation on off-farming probability. Based on the estimates of the seemingly unrelated regression, rural households with an ill-healthy breadwinner have an average 0.042 percentage points higher probability working off-farming locally (the direct effect) compared to their counterparts. The effect will further be intensified through farming. The magnitude of this indirect effect is about 0.004%, which is the multiplication of an illness induced marginal increase in farming participation (0.044%) and a farming appreciation induced marginal increase in local off-farming participation (0.081%). The sum of the direct and indirect effects (0.046%) equals the total effect calculated by the Logit model (0.046%) and is pretty much the same with the Probit model (0.047%). This mediating role of farming is also verified in the effect of chronic illness on migration. While the direct effect of illness on migration is insignificant, ill-health induced farming appreciation significantly depresses the probability of migration. Comparatively, health-impaired rural households tend to diversify toward local off-farm employments instead of migration as a complementary strategy for farming.

### 4.3. Income Enhancement

Table 4 shows that more assets and less debt are significantly associated with more diverse income generation strategies. The estimated predictions of diversification are included in the income equations to mitigate potential endogeneity bias. Coefficients in column (11) reflect resilience capacities of rural livelihood diversification without considering the impact of health shocks. The positive and significant estimator indicates that diversification significantly enhances income growth of rural households. We further add a square term of diversification in column (12) to check whether there is a U-shaped, an inverse U-shaped, or another pattern of relationship exists between diversification and income. We observe a concave down income returns curve with respect to diversification at 1.30 whereas the normalized Herfindahl–Simpson income diversification index falls to the range of 0 to 1. That is, without considering health status of the breadwinner, the relationship between diversification and income in rural China is better categorized as upward sloping with a diminishing effect rather than being the inverse U-shape.

**Figure 1:**
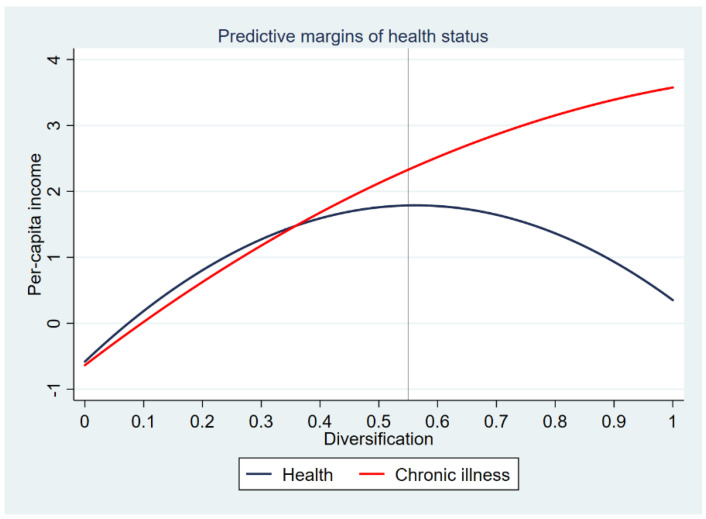
Income returns to diversification by health status.

We are particularly interested in the role of diversification for mitigating the impact of health shocks experienced by rural households. Chronic illness is significantly related to livelihood choices and household income. Our model confirms an on average 11% lower per capita income for rural households with a health-impaired breadwinner, which is quite robust irrespective of the relationship between diversification and income specified (columns (11) and (12)). The effect of resilience capacities in mitigating the impact of health shocks is given by the estimated coefficients of the interactions of diversification variables and chronic illness in column (13). Livelihood diversification per se does not have a significant mitigating effect on the income impact of chronic illness whereas its squared term contributes significantly to reduce the negative effect of ill health on household income. One of the explanations is that the resilience capacity of diversification attenuates when it approaches 0, i.e., specialization. Figure 1 depicts the predictive margins for health and chronic illness with respect to the levels of diversification. It can be seen that both health conditions respond more similarly with diversification below 0.4, and the income of households with a health-impaired breadwinner has an increasing sharper reaction to diversification as it approaches 1. In the case of healthy farmers, we obtain a traditional converse U-shaped relationship between diversification and income. For the health-impaired, there is evidence in favor of an upward sloping with diminishing marginal response curve, which explains the diversification-income relationship we observed in column (12). 

### 4.4. Effects of the Controls

The coefficients estimated that the partial relationships between livelihood choices and income and further demographic and economic fundamentals by and large meet with theoretical expectations. We find that age of the breadwinner has a significant and positive effect on farming probability and significant but negative effects on both local off-farming and migrating probabilities. As expected, per capita household income decreases with the age of breadwinner. Household size acts as a positive driver of farming and migration while it has a significantly negative effect on local off-farming participation. Households with larger populations also diversify more, though their per capita income is lower. Number of the youth has effects quite opposite those of household size. The probabilities of farming and migration decrease with the number of the youths while the probability of local off-farm employment increases. One more youth decreases diversification significantly by 0.042. Land area has significantly negative effects on farming and migrating probabilities whereas Probit and Logit regressions report negative but insignificant marginal effects. The level of diversification significantly decreases with the area of land while per capita income appreciates strongly in the presence of more arable land. Subsistence allowance and agricultural subsidy both promote diversification as they may help relax liquidity constraints. In line with policy provisions, we find a significant and negative partial association between per capita income and subsistence allowance while its relationship with agricultural subsidy is negative but insignificant. This is because that subsistence allowance targets at relief for the extremely poor in terms of food, clothing, medical care, housing and funeral expenses, while agricultural subsidy is direct treasury transfer to all farmers and accounts for a relatively low percentage of revenue.

### 4.5. Robustness Check

The morbidity rate of chronic illness of our sample is much higher than these detected in the early years and other rural areas such as a 19.2% among Liuyang rural residents in 2007 [41]. While health inequality may be intensified as the rural healthy workforce permanently migrates to urban areas, self-reported diagnosis may also be overstated, as we did not ask for diagnosis certification (i.e., from the town clinic or superior level hospitals) during interview. In this section, we consider two alternative measures of chronic illness. The first is a factual measure that is a composite of self-reported diagnosis and medical cost. The dummy indicator of illness equals one if the breadwinner had been diagnosed with at least one chronic disease and his or her medical expenditure was not zero in the year before interview. The second is an inpatient measure that is a composite of self-reported diagnosis and inpatient treatment. The breadwinner with self-reported chronic illness is defined as health impaired if he or she had inpatient treatment in the year before interview. The advantage of the composites over the self-reported diagnosis is that they correct for the possible overestimation due to measurement error. Both measures substantially decrease the percentage of ill-healthy breadwinner in our sample, with 59.82% reported by the factual indicator and 36.52% the inpatient indicator.

Table 5 presents regression results for these two composite indicators. The same methodology as above is applied. In general, the results show the same trend concerning the relationship between illness and livelihood choices, and diversification and income. A significant and positive effect of ill health on farming probability is verified using both measures and farming participation in turn acts as a significant driver for local off-farm employments while it impedes working migration. In line with expectations, ill health has a directly positive effect on local off-farming probability while a directly negative effect on migration after controlling farming participation. One exception is that both the effects are with plausible signs but statistically insignificant when we adopt the inpatient indicator. However, since the estimated partial effect of ill health on farming is much higher compared to our benchmark model, its indirect effect on off-farming probabilities is amplified through farming decision and the total effect is almost the same in magnitude across all models. 

Both indicators support an upward sloping relationship between diversification and income whereas the mediating effect of diversification on the income returns of health is statistically insignificant. Recall that, with self-reported diagnosis, we observed an inverse U-shaped relationship between diversification and income for rural households with a healthy breadwinner. That is to say, health measures exaggerating impairment may lead to a less sharp income response to diversification. After controlling the mediating effect, we observe a negative but insignificant effect of ill health on household income in both models, which is consistent with the result of self-reported diagnosis.

## 5. Discussion and Conclusions

How rural livelihood is affected by chronic illness is of critical relevance for households and policy makers. In addition to occupation and productivity changes, an often overlooked fact is that rural livelihood consists in portfolio adjustments especially with the growth of off-farming opportunities. Chronic illness can unfold heterogeneous impacts across livelihood strategies and such rural welfare, an issue that has so far been largely ignored by the literature.

Estimating a seemingly unrelated trivariate model with household-level data for different livelihood choices from rural China, the results of this paper demonstrate evidence of positive effects of chronic illness on farming and local off-farming probabilities while they show a negative effect on migrating probability. This holds after controlling for equation correlations, county fixed effects and possibly the endogeneity of livelihood decisions. We additionally show that the heterogeneous effects might be explained by strategy complementarity under the grading reimbursement system of the Cooperative Medical Scheme in rural China. Breadwinners with chronic illness might be constrained to farming for the higher reimbursement of local healthcare, and local off-farming employment is a complementary strategy to farming for communication and accommodation cost reduction. Further analyses show a pro-illness characteristic of diversification associated with sharper increases in per capita household income.

Additionally, our estimates suggest that projected severe health problems will be a huge hampering factor to poverty reduction in rural China if the health impaired continue to rely on farming and local off-farming employment. As with any study focusing on area deprivation, it is evident that the estimates on the demography–livelihood relationship have to be treated with appropriate care in terms of health problems. In this study, it is proposed that rural households design livelihood strategies to account for the constraints imposed by exposures to health risks. The separation of farming from non-farming and local from migrating livelihood strategies, then, makes sense if one considers coping responses to versus challenges of chronic illness. As seen above, diversifying towards farming and local off-farming employments, and especially their combination, helps rural households mitigating the adverse effects of ill health on income. Alternatively, one may elect to focus on where the barrier happens, rather than which coping strategy is adopted. In this case the migrating impacts become particularly important, as people who are willing to and can work may be less supported in an urban labor market due to a variety of health reasons.

Concerning the economic magnitude of the results, the heterogeneity in each of the household strategies reflects differences in activity returns between breadwinners with different health levels. The most obvious implications of these findings for policy are related to the field of health shocks and rural livelihood recovery: policy interventions enhancing a pro poor-health rural economy might gain higher importance compared to facilitating migration in health-related poverty reduction. As agribusiness moves quickly to catch up with the eCommerce trend, labor-intensive agriculture provides high potential for local employment growth [42]. An agriculture-for-development approach calls for government to increase financial assistance and opportunities in the rural economy while enhance skills to allow the unhealthy with no work limitations to seize these opportunities. For those with work limitations, improving productivity in subsistence agriculture can allow them to secure their food consumption and health. In the interim, their greatest needs are for yield-stabilizing technologies and resilient farming systems [43]. 

While income enhancement provides a useful insight into the economic benefits of diversification, it does not provide further information on economies of scope due to data limitations. Future empirical research can address the efficiency of livelihood complementarities from the perspective of cost reduction of different diversification schemes [44]. To this end, data on rural labors specializing in different occupations are needed. Besides, it must be noted that local off-farm employments must not be the primary complementary strategy to farming for health-impaired populations. Since the efficiency of diversification relies on the managerial behavior assumption of farmers, in this article we use the concept of complementarity as an indicator for the diversification strategy. If productivity maximization of the health impaired is on the policy agenda, the interaction effect of health and diversification on total factor productivity should be considered, which relies on more detailed data and is subject to further research.

## Figures and Tables

**Table 1:** Variables used in model specification.

Variable	Definition
Dependent variables	
On-farm	Dummy: 1 if farming is a livelihood strategy for the household in 2016; 0 otherwise
Local off-farm	Dummy: 1 if local non-farm employment is a livelihood strategy for the household in 2016; 0 otherwise
Migrant	Dummy: 1 if migration is a livelihood strategy for the household in 2016; 0 otherwise
Income	Per capita income: ratio of total household income (sum of farm income, local non-farm income, migrating income and other income) to household size (10,000 RMB)
Instruments	
poverty	Dummy: 1 if the household resides in a government designated poor village; 0 otherwise
assets	Counted number of household owned refrigerators, air conditioners, washers, computers, televisions, motorcycles, and cars
debt	Total accumulated household debt before 2016 (10,000 RMB)
Variables of interest and controls	
di	Normalized Herfindahl-Simpson income diversification index
illness	Dummy: 1 if the household breadwinner had been diagnosed with at least one chronic disease before 2016; 0 otherwise
age	Age of the breadwinner
size	Number of household members
youth	Number of household members aged below 16
land	Area of arable land for rice production (mu, 1/15 hectare)
sub-all	Subsistence allowance for food, clothes, medical care, housing and death related in 2016 (10,000 RMB)
sub-agr	Agricultural subsidy in 2016 (10,000 RMB)

**Table 2:** Summary of variables by health status.

	Illness = 1		Illness = 0		Difference
	Mean	Std Err	Mean	Std Err	
On-farm	0.546	0.009	0.529	0.018	0.017
Local off-farm	0.428	0.009	0.395	0.017	0.033 *
Migrant	0.227	0.008	0.269	0.016	−0.041 **
Income	0.926	0.015	1.086	0.033	−0.161 ***
poverty	1.809	0.007	1.792	0.015	0.017
assets	2.242	0.027	2.424	0.054	−0.182 ***
debt	0.793	0.107	0.702	0.087	0.090
di	0.284	0.005	0.287	0.010	−0.003
age	55.031	0.231	51.084	0.474	3.947 ***
size	2.875	0.026	3.039	0.051	−0.164 ***
youth	0.365	0.013	0.572	0.030	−0.207 ***
land	5.412	3.866	1.924	0.641	3.489
sub-all	0.287	0.004	0.222	0.009	0.065 ***
sub-agr	0.050	0.002	0.056	0.004	−0.006

*** *p* < 0.01, ** *p* < 0.05, * *p* < 0.1.

**Table 3:** Chronic illness and livelihood strategies.

	Probit			Logit			Seemingly Unrelated Regression		
	On-Farm	Local Off-Farm	Migrant	On-Farm	Local Off-Farm	Migrant	On-Farm	Local Off-Farm	Migrant
	(1)	(2)	(3)	(4)	(5)	(6)	(7)	(8)	(9)
On-farm							---	0.081 ***	−0.153 ***
								(0.016)	(0.014)
poverty	0.045 **	0.019	−0.011	0.045 **	0.019	−0.011	0.044 **	---	---
	(0.021)	(0.021)	(0.018)	(0.021)	(0.021)	(0.018)	(0.021)		
illness	0.043 **	0.047 **	−0.030 *	0.043 **	0.046 **	−0.029 *	0.044 **	0.042 **	−0.023
	(0.020)	(0.020)	(0.017)	(0.020)	(0.020)	(0.017)	(0.020)	(0.020)	(0.017)
age	0.002 ***	−0.003 ***	−0.003 ***	0.002 ***	−0.003 ***	−0.003 ***	0.002 ***	−0.003 ***	−0.003 ***
	(0.001)	(0.001)	(0.001)	(0.001)	(0.001)	(0.001)	(0.001)	(0.001)	(0.001)
size	0.069 ***	−0.058 ***	0.047 ***	0.068 ***	−0.058 ***	0.046 ***	0.068 ***	−0.061 ***	0.058 ***
	(0.007)	(0.007)	(0.006)	(0.007)	(0.007)	(0.006)	(0.007)	(0.007)	(0.006)
youth	−0.035 ***	0.093 ***	−0.027 ***	−0.033 ***	0.093 ***	−0.026 ***	−0.034 ***	0.093 ***	−0.032 ***
	(0.013)	(0.013)	(0.011)	(0.013)	(0.013)	(0.010)	(0.013)	(0.012)	(0.011)
land	−0.000	0.001	−0.000	−0.000	0.001	−0.000	−0.000 *	0.000 **	−0.000 ***
	(0.000)	(0.001)	(0.001)	(0.000)	(0.001)	(0.001)	(0.000)	(0.000)	(0.000)
sub-all	−0.059 *	0.109 ***	−0.045	−0.062 *	0.111 ***	−0.043	−0.056 *	0.113 ***	−0.061 **
	(0.034)	(0.034)	(0.030)	(0.034)	(0.034)	(0.030)	(0.034)	(0.034)	(0.030)
sub-agr	0.538 ***	−0.029	0.065	0.610 ***	−0.035	0.067	0.480 ***	−0.070	0.142 *
	(0.103)	(0.091)	(0.077)	(0.119)	(0.091)	(0.073)	(0.090)	(0.090)	(0.078)
Regions	Yes	Yes	Yes	Yes	Yes	Yes	Yes	Yes	Yes
(Pseudo) R2	0.119	0.095	0.093	0.119	0.095	0.092	0.155	0.128	0.126

Standard errors in parentheses. *** *p* < 0.01, ** *p* < 0.05, * *p* < 0.1.

**Table 4:** Diversification and income enhancement by groups.

	Diversification	Income		
	(10)	(11)	(12)	(13)
assets	0.017 ***	---	---	---
	(0.003)			
debt	−0.001 ***	---	---	---
	(0.000)			
di	---	5.279 ***	6.741 ***	7.612 ***
		(0.657)	(0.846)	(1.449)
di^2^	---	---	−2.601 ***	−5.445 ***
			(0.876)	(2.179)
di*illness	---	---	---	−0.935
				(1.088)
di^2^*illness	---	---	---	3.190 *
				(1.668)
illness	0.006	−0.110 ***	−0.109 ***	−0.131
	(0.011)	(0.037)	(0.037)	(0.178)
poverty	−0.007	0.087 **	0.087 **	0.087 **
	(0.012)	(0.035)	(0.035)	(0.035)
age	−0.000	−0.008 ***	−0.008 ***	−0.008 ***
	(0.000)	(0.001)	(0.001)	(0.001)
size	0.047 ***	−0.248 ***	−0.240 ***	−0.237 ***
	(0.004)	(0.037)	(0.038)	(0.037)
youth	−0.042 ***	0.051	0.043	0.039
	(0.007)	(0.033)	(0.033)	(0.033)
land	−0.000 ***	0.000 ***	0.000 ***	0.000 ***
	(0.000)	(0.000)	(0.000)	(0.000)
sub-all	0.037 *	−0.512 ***	−0.525 ***	−0.532 ***
	(0.020)	(0.066)	(0.066)	(0.066)
sub-agr	0.083 *	−0.176	−0.179	−0.188
	(0.048)	(0.148)	(0.148)	(0.148)
Regions	Yes	Yes	Yes	Yes
Robust score $\chi^{2}$	64.127 ***	---	---	---
Robust Regression F	58.831 ***	---	---	---
Overidentification $\chi^{2}$	1.183	---	---	---
R-squared	0.168	0.165	0.167	0.175

States interaction between two variables (see Equation (2)). Robust standard errors in parentheses. *** *p* < 0.01, ** *p* < 0.05, * *p* < 0.1.

**Table 5:** Robustness check results.

	The Factual Indicator				The Inpatient Indicator			
Panel A: Health and livelihood strategies								
	Probability				Probability			
	On-farm	Local off-farm		Migrant	On-farm	Local off-farm		Migrant
On-farm	---	0.079 ***		−0.153 ***	---	0.081 ***		−0.154 ***
		(0.016)		(0.014)		(0.016)		(0.014)
poverty	0.043 **	---		---	0.040 *	---		---
	(0.021)				(0.021)			
illness	0.050 ***	0.046 ***		−0.029 **	0.082 ***	0.007		−0.002
	(0.016)	(0.016)		(0.014)	(0.016)	(0.016)		(0.014)
Panel B: Diversification and income enhancement								
	Diversification	Income			Diversification	Income		
		(a)	(b)	(c)		(a)	(b)	(c)
assets	0.017 ***	---	---	---	0.017 ***	---	---	---
	(0.003)				(0.003)			
debt	−0.001 ***	---	---	---	−0.001 ***	---	---	---
	(0.000)				(0.000)			
di	---	5.356 ***	6.810 ***	7.212 ***	---	5.346 ***	6.837 ***	6.921 ***
		(0.667)	(0.853)	(1.072)		(0.668)	(0.855)	(1.072)
di^2^	---	---	−2.593 ***	−3.548 ***	---	---	−2.657 ***	−2.949 ***
			(0.873)	(1.341)			(0.870)	(1.118)
di*illness	---	---	---	−0.391	---	---	---	0.088
				(0.821)				(0.747)
di^2^*illness	---	---	---	1.108	---	---	---	0.212
				(1.214)				(1.073)
illness	0.007	−0.118 ***	−0.116 ***	−0.108	0.007	−0.073 **	−0.072 ***	−0.119
	(0.009)	(0.031)	(0.031)	(0.137)	(0.009)	(0.030)	(0.030)	(0.130)

States interaction between two variables (see Equation (2)). Standard errors in parentheses. *** *p* < 0.01, ** *p* < 0.05, * *p* < 0.1.

## Data Availability

The data presented in this study are available upon the request from the corresponding author.

## References

[1] Mishra A.K., Morehart M.J. (2001). Off-farm investment of farm households: A Logit Analysis. Agric. Financ. Rev..

[2] Broeck G.V., Kilic T. (2019). Dynamics of off-farm employment in Sub-Saharan Africa: A gender perspective. World Dev..

[3] Zhang L., Dong Y., Liu C., Ba Y. (2018). Off-farm employment over the past four decades in rural China. China Agric. Econ. Rev..

[4] Ellis F. (2000). Rural Livelihoods and Diversity in Developing Countries.

[5] Curie J., Madrian B., Ashenfelter O., Card D. (1999). Health, health insurance, and the labor market. Handbook of Labor Economics.

[6] Cai L.X., Kalb G. (2006). Health status and labour force participation: Evidence from Australia. Health Econ..

[7] Rodriguez-Alvarez A., Rodriguez-Gutierrez C. (2018). The impact of health on wages: Evidence for Europe. Eur. J. Health Econ..

[8] Pond R., Stephens C., Alpass F. (2010). How health affects retirement decisions: Three pathways taken by middle-older aged New Zealanders. Ageing Soc..

[9] Davis B., Giuseppe S.D., Zezza A. (2017). Are African households (not) leaving agriculture? Patterns of households’ income sources in rural Sub-Saharan Africa. Food Policy.

[10] Falco P., Haywood L. (2016). Entrepreneurship versus joblessness: Explaining the rise in self-employment. J. Dev. Econ..

[11] Abdulai A., CroleRees A. (2001). Determinants of income diversification amongst rural households in Southern Mali. Food Policy.

[12] Schwarze S., Zeller M. (2005). Income diversification of rural households in Central Sulawesi, Indonesia. Q. J. Int. Agric..

[13] Dedehouanou S.N., Mcpeak J.G. (2020). Diversify more or less? Household income generation strategies and food security in rural Nigeria. J. Dev. Stud..

[14] Démurger S., Fournier M., Yang W. (2010). Rural households decisions towards income diversification—Evidence from a township in northern China. China Econ. Rev..

[15] Zhao J., Barry P.J. (2014). Income diversification of rural households in China. Can. J. Agric. Econ..

[16] Zhao J. (2014). Rural income diversification patterns and their determinants in China. Agric. Econ..

[17] Djido A.I., Shiferaw B.A. (2018). Patterns of labor productivity and income diversification—Empirical evidence from Uganda and Nigeria. World Dev..

[18] Leng C.X., Ma W.L., Tang J.J., Zhu Z.K. (2020). ICT adoption and income diversification among rural households in China. Appl. Econ..

[19] Davis J., Bennett R. (2007). Livelihood adaptation to risk: Constraints and opportunities for pastoral development in Ethiopia’s afar region. J. Dev. Stud..

[20] Chambers R.G., Voica D.C. (2017). “Decoupled” farm program payments are really decoupled: The theory. Am. J. Agric. Econ..

[21] Kelsey T. (1991). Fatal farm accidents in New York: Estimates of their costs. Agric. Resour. Econ. Rev..

[22] Loureiro M. (2009). Farmers’ health and agricultural productivity. Agric. Econ..

[23] Ulimwengu J. (2009). Farmers’ health and agricultural productivity in rural Ethiopia. Afr. J. Agric. Resour. Econ..

[24] Audibert M., Etard J. (2003). Productive benefits after investment in health in Mali. Econ. Dev. Cult. Chang..

[25] Fink G., Masiye F. (2015). Health and agricultural productivity: Evidence from Zambia. J. Health Econ..

[26] Hard D.L., Myers J.R., Snyder K.A., Casini V.J., Morton L.L., Cianfrocco R., Fields J.K. (1999). Identifying work-related fatalities in the agricultural production sector using two national occupational fatality surveillance systems, 1990–1995. J. Agric. Saf. Health.

[27] Pauly M., Zweifel P., Scheffler R., Preker A., Bassett A. (2006). Private health insurance in developing countries. Health Aff..

[28] Drechsler D., Jutting J. (2005). Private health insurance for the poor in developing countries. Policy Insights.

[29] Thomas V. (2009). Health care in developing countries-need for finance, education or both?. Calicut Med. J..

[30] Ahearn M., El-Osta H., Dewbre J. (2006). The impact of coupled and decoupled government subsidies on off-farm labor participation of US farm operators. Am. J. Agric. Econ..

[31] Mishra A.K., Chang H.H. (2012). Can off farm employment affect the privatization of social safety net? The case of self-employed farm households. Food Policy.

[32] China SCI (2014). China 2013 Human Rights Report. The State Council Information Office of the People’s Republic of China. http://www.scio.gov.cn/zfbps/ndhf/2014/Document/1373162/1373162_1.htm.

[33] Liao P.A., Taylor J.E. (2010). Health care reform and farm women’s off-farm labor force participation: Evidence from Taiwan. J. Agric. Resour. Econ..

[34] Zhou X.S., Ma W.L., Renwick A., Li G.C. (2020). Off-farm work decisions of farm couples and land transfer choices in rural China. Appl. Econ..

[35] Wilde J. (2000). Identification of multiple equation probit models with endogenous dummy regressors. Econ. Lett..

[36] Filippou P., Marra G., Radice R. (2017). Penalized likelihood estimation of a trivariate additive Probit model. Biostatistics.

[37] Johny J., Wichmann B., Swallow B.M. (2017). Characterizing social networks and their effects on income diversification in rural Kerala, India. World Dev..

[38] Block S., Webb P. (2001). The dynamics of livelihood diversification in post-famine Ethiopia. Food Policy.

[39] Babatunde R.O., Qaim R. (2009). Patterns of income diversification in rural Nigeria: Determinants and impacts. Q. J. Int. Agric..

[40] Wooldridge J.M., Rao C.R., Maddala G.S., Phillips P.C.B., Srinivasan T.N. (1995). Score diagnostics for linear models estimated by two stage least squares. Advances in Econometrics and Quantitative Economics: Essays in Honor of Professor.

[41] Huang X., Chen M.S., Tan H.Z., Xiao S.Y., Deng J. (2013). The morbidity rate of chronic disease among Chinese rural residents: Results from Liuyang cohort. Med. Princ. Pract..

[42] Pu M.Z., Zhong Y. (2020). Rising concerns over agricultural production as COVID-19 spreads: Lessons from China. Glob. Food Secur..

[43] World Bank (2007). World Development Report 2008: Agriculture for Development.

[44] Wimmer S., Sauer J. (2020). Diversification economies in dairy farming—Empirical evidence from Germany. Eur. Rev. Agric. Econ..

